# Loss of Surfactant Protein A Alters Perinatal Lung Morphology and Susceptibility to Hyperoxia-Induced Bronchopulmonary Dysplasia

**DOI:** 10.3390/antiox13111309

**Published:** 2024-10-28

**Authors:** Shaili Amatya, Matthew Lanza, Todd M. Umstead, Zissis C. Chroneos

**Affiliations:** 1 Department of Pediatrics, Division of Neonatal-Perinatal Medicine, Pulmonary Immunology and Physiology Laboratory, Pennsylvania State University College of Medicine, Hershey, PA 17033, USA; tumstead2@pennstatehealth.psu.edu (T.M.U.); zchroneos@pennstatehealth.psu.edu (Z.C.C.); 2 Department of Comparative Medicine, Pennsylvania State University College of Medicine, Hershey, PA 17033, USA; mlanza1@pennstatehealth.psu.edu; 3 Department of Microbiology and Immunology, Pennsylvania State University College of Medicine, Hershey, PA 17033, USA

**Keywords:** bronchopulmonary dysplasia, hyperoxia, surfactant protein A (SP-A), prematurity, neonatal

## Abstract

Bronchopulmonary dysplasia (BPD) is a condition of poor alveolar formation that causes chronic breathing impairment in infants born prematurely. Preterm lungs lack surfactant and are vulnerable to oxidative injuries driving the development of BPD. Our recent studies reported that surfactant protein A (SP-A) genetic variants influence susceptibility to neonatal lung disease. SP-A modulates activation of alveolar macrophages and parturition onset in late gestation. We asked whether a lack of SP-A alters alveolarization in a mouse model of hyperoxia-induced BPD. SP-A-deficient and control newborn mice were exposed to either clinically relevant 60% O_2_ hyperoxia or normoxia for 5–7 days. Alveolar formation was then assessed by mean linear intercept (MLI) and radial alveolar count (RAC) measurements in lung tissue sections. We report that the combination of SP-A deficiency and hyperoxia reduces alveolar growth compared to WT mice. The morphometric analysis of normoxic SP-A-deficient lungs showed lower RAC compared to controls, indicating reduced alveolar number. In the presence of hyperoxia, MLI was higher in SP-A-deficient lungs compared to controls. Differences were statistically significant for female pups. Spatial proteomic profiling of lung tissue sections showed that hyperoxia caused a 4-fold increase in the DNA damage marker γH2Ax in macrophages of SP-A-deficient lungs compared to normoxia. Our short report suggests an important role for SP-A in perinatal lung development and the protection of lung macrophages from oxidant injury. These studies warrant future investigation to discern the temporal interaction of SP-A, gender, oxidant injury, and lung macrophages in perinatal alveolar formation and development of BPD.

## 1. Introduction

Bronchopulmonary dysplasia (BPD) is the most common chronic respiratory morbidity of prematurity. BPD is characterized by an arrest in lung growth and disruption of alveolar development [1]. BPD is associated with high mortality, increased healthcare costs, and poor neurodevelopmental outcomes [2]. Despite advances in critical care, there is increasing BPD incidence with challenges in developing newer diagnostics and therapeutics. The innate immune system likely plays an important role in BPD pathogenesis in the setting of inflammation and oxidative stress [3]. Premature neonates are born with surfactant deficiency and lack immune protection by surfactant protein A (SP-A). SP-A plays a dual role in surfactant ultrastructure and modulation of inflammation and innate host defense by alveolar macrophages (AMs) [4]. SP-A gene polymorphisms have been associated with an increased risk for the development of BPD in preterm infants [5,6]. SP-A is not incorporated in commercially available surfactant replacement therapies that are used to treat preterm infants at risk of BPD.

SP-A is expressed and secreted by alveolar type II epithelial cells of the fetal lung in late gestation. SP-A modulates the activation of AMs and the onset of parturition in late gestation [7,8,9]. SP-A mediates bacterial phagocytosis [10] and secretion of inflammatory mediators by macrophages [11]. These SP-A functions are relevant to BPD pathogenesis as infection and inflammation play an important role in causing lung injury. Additionally, SP-A affects the NAD(H) redox status of AMs in response to ozone exposure [12]. Oxidative stress due to hyperoxia is an important factor in the pathology of BPD [3]. Here, we report that lack of SP-A affects neonatal lung growth in response to hyperoxia in SP-A-deficient mice.

## 2. Materials and Methods

### 2.1. Mice 

All animal work was approved by the Institutional Animal Care and Use Committee (Protocol No 101786) and performed within an American Association for the Accreditation of Laboratory Animal Care-certified barrier facility at Pennsylvania State University College of Medicine. Mice were housed under specific pathogen-free conditions in micro-isolator cages, provided food and sterile water ad libitum, and maintained on a 12 h-light/12 h-dark cycle. The generation of SP-A-deficient mice has been described previously [13]. SP-A-deficient mice were backcrossed to the C57BL/6J background [14]. WT male and female C57BL/6J controls were purchased from JAX labs at 4–6 weeks of age and bred in house. Male and female mice were used for mating at the age of 6–8 weeks at a ratio of 1:1 male to female.

### 2.2. Exposure to Hyperoxia or Normoxia

Dams and pups were exposed to either hyperoxia (60% O_2_) or maintained at normoxia in room air (21% O_2_) from postnatal day 1 (P1) until postnatal day 5–7 (P5–P7). The litter size was maintained at 6–10 pups per exposure. Nursing dams received food ad libitum. If nursing dams were unable to nurse due to oxygen toxicity, they were transferred between room air and hyperoxia. Dams did not exhibit overt signs of illness at 60% O_2_ during the 7-day exposure period.

### 2.3. Histology and Morphometry

Mice were anesthetized by intraperitoneal injection of Ketamine/Xylazine (90/10 mg/kg/dose) followed by laparotomy and exsanguination via inferior vena cava incision. The lungs were then exposed via thoracotomy, intubated using a tracheal cannula, and fixed with 10% (*v*/*v*) formalin or 4% (*w*/*v*) paraformaldehyde at 25 cm H_2_O hydrostatic pressure. The fixed lungs were then processed for paraffin embedding, sectioned at 6 μm, and stained with the periodic acid Schiff (PAS) stain. Sections were evaluated blinded to the genotype. The quantitative measures included mean linear intercept (MLI) and radial alveolar count (RAC). MLI was calculated by determining the average distance between the intersections of alveolar septal tissue. To achieve this, we used a stereological method that involves randomly placing a counting grid over the lung tissue. The number of times the grid intersects the alveolar septal tissue was counted, and the average distance between these intersections was computed to obtain the MLI. RAC was determined by counting the number of alveoli between the pleural surface of the lung and the nearest terminal bronchiole. We employed a systematic sampling method to determine RAC, which involves counting the number of alveoli in a series of parallel lines intersecting the lung parenchyma. RAC was expressed as the number of alveoli per unit length of the line, and it provides a measure of the density of alveoli in the lung tissue.

### 2.4. Spatial Proteomic Profiling by GeoMx^®^ Nanostring

The changes in CD11b+ and ABAC3+ populations in the lungs exposed to hyperoxia and normoxia were assessed using Nanostring spatial proteomics. Two representative slides of mouse lungs from SP-A knockout male pups were analyzed. A total of 24 AOI (area of interest) with 8 CD11b+, 8ABCA3+, and 8DNA+ populations were assessed. The mouse immune-oncogene core protein panel plus immune activation status, immune cell typing, cell death, MAPK signaling, PI3k/AKT signaling, and pan-tumor panels that included 72 protein targets and 6 housekeeping targets were analyzed. The morphology markers that were used were αSMA, CD11b, ABCA3, and DAPI to visualize airway smooth muscle and blood vessels, macrophages, alveolar type II epithelial cells, and DNA, respectively. The gene expression patterns were assessed by the DSP data analysis tool (GeoMx^®^ Nanostring, Seattle, WA, USA). Data normalization for the DSP protein assay was performed by normalizing to housekeeping protein controls—GAPDH, Histone H3, S6 and scaling to isotype negative controls- Rat IgG2a, Rat IgG2b, Rabbit IgG. Housekeeping and isotype control normalization were used to control for within-slide differences in the region of interest (ROI) cellularity and between-slide variability (differences in signal volume due to tissue quality, fixation, processing, etc.). Further normalization was performed by scaling to the area to normalize the geomean of the masked area to correct for variable surface area, and scaling to nuclei was performed to normalize the geomean of nuclei count of masked ROI.

### 2.5. Immunohistochemistry

Immunofluorescent staining of lung tissues was performed on formalin-fixed paraffin-embedded sections. The sections were washed in xylene and gradually rehydrated in ethanol (100%, 95%, 70%, 50%) and processed per the modified Chapman protocol. Samples were washed with Phosphate Buffered Saline (PBS), blocked with 1% BSA/5% horse serum (45 min, room temperature), and then incubated overnight at 4°C with primary antibody (1:500 dilutions in 0.5% BSA), washed 3 times with PBS, incubated with appropriate fluorescent-labeled secondary antibodies (1:1000 dilution in 0.5% BSA, Life Technologies Inc., Carlsbad, CA, USA) and the nuclear marker, 4′,6-diamidino-2-phenylindole, dihydrochloride (DAPI, Invitrogen cat# P36962). The primary antibody used was γH2AX (Cell Signaling #9718). Slides were imaged using fluorescent microscopy (Nikon Eclipse TE2000-U). Fluorescence intensity was quantified and analyzed via ImageJ software (ver 1.54, Wayne Rasband, NIH, USA) [15].

### 2.6. Statistical Analysis

Descriptive characteristics were computed as the means +/− standard deviation (SD). Two-way ANOVA was used to compare groups using GraphPad Prism software, ver 10.1.2 All tests were 2 tailed, and significance was assigned for *p* < 0.05. The *p*-value was adjusted using Tukey’s multiple comparisons test. There were 3–10 mouse pups per sex per group.

## 3. Results

### 3.1. Lack of SP-A Enhances Hyperoxia-Induced Arrest in Alveolar Formation

**Figure 1:**
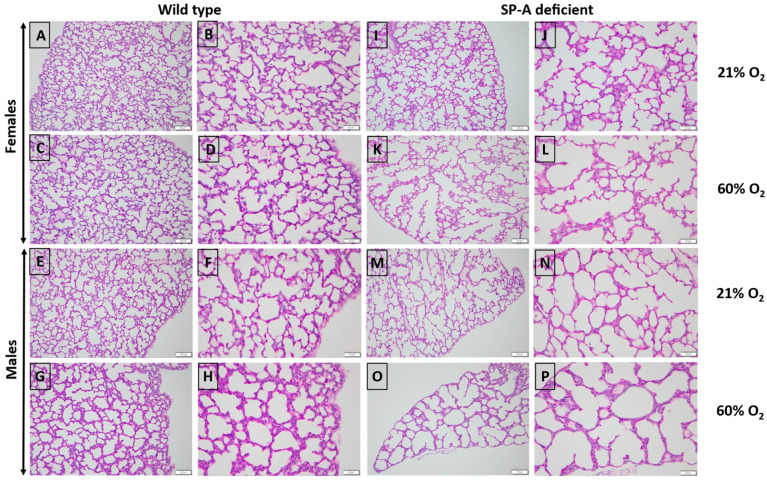
Prominent hyperoxia-induced alveolar simplification in SP-A-deficient lungs. Representative images of PAS-stained formalin-fixed lungs from WT (**A**–**H**) and SP-A-deficient (**I**–**P**) pups exposed to normoxia (**A**–**D**,**I**–**L**) or hyperoxia (**E**–**H**,**M**–**P**). Images were captured at 200× (100 µm- scale bar) (left columns) or 400× (50 µm- scale mar)(right columns) magnification from female and male pups, as indicated. All images were captured on postnatal day 7 except images (**I**,**J**), which were captured on day 6.

**Figure 2:**
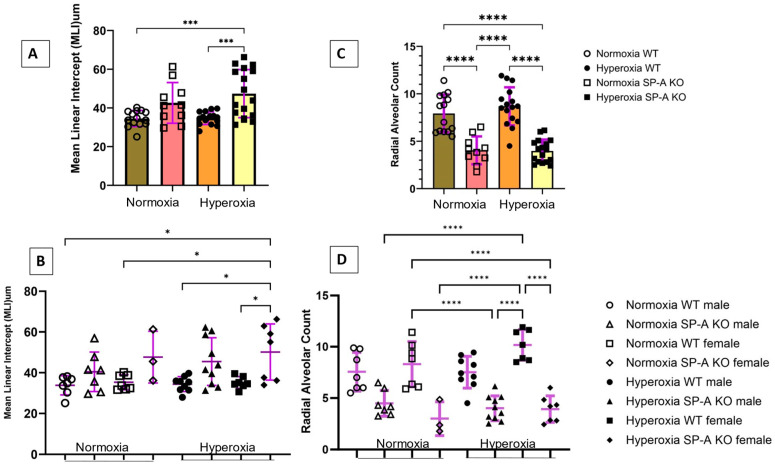
Lack of surfactant protein A (SP-A) modulates hyperoxia-induced alveolar simplification. Neonatal pups were exposed to either normoxia or hyperoxia with oxygen exposure at 60% from birth till postnatal day 5–7. Lack of SP-A causes worsening of alveolar simplification secondary to hyperoxia as noted by increased mean linear intercept (**A**) and reduced radial alveolar count (**C**) compared to wild type. Lack of SP-A noted to contribute to sex differences in hyperoxia-induced neonatal lung injury, as female SP-A knockout (KO) mice are more prone to alveolar simplification (**B**,**D**) when exposed to hyperoxia when compared to wild type (WT) genotype. Data are shown as the means ± SD. *N* = 3–10 mouse pups per sex per group. * adjusted *p* < 0.05, *** adjusted *p* < 0.001, **** adjusted *p* < 0.0001.

To assess the impact of SP-A deficiency in alveolar simplification, we exposed mice to 60% O_2_ for 5–7 days after birth when the saccular to alveolar transition of alveolar development is known to occur [16]. This period corresponds to 24–36 weeks gestation of human neonates when premature infants are at high risk of developing BPD. The 60% O_2_ dose is comparable to clinical settings and sufficient to induce alveolar arrest in WT mouse lungs [17]. We used MLI and RAC as hallmark morphometric measures of alveolar development and growth, respectively [18,19]. Data were pooled together or analyzed separately for male and female pups (Figure 1). Hyperoxia resulted in a significant increase in MLI to 47.4 ± 12.4 µm (Mean ± SD) in SP-A-deficient lungs compared to 34.7 ± 2.3 µm (adjusted *p* = 0.0005) and 34.5 ± 4.1 µm (adjusted *p* = 0.0006) in hyperoxic and normoxic WT lungs, respectively (Figure 2A). The SP-A-deficient female lungs were the most sensitive to hyperoxia with an MLI at 50.1 ± 13.8 µm compared to 33.8 ± 4.7 µm (adjusted *p* = 0.018) in normoxic WT male lungs, 35.3 ± 3.7 µm (adjusted *p* = 0.043) in normoxic WT female lungs, 34.4 ± 3.6 µm (adjusted *p* = 0.014) in hyperoxic WT male lungs, and 35.14 ± 2.9 µm (adjusted *p* = 0.039) in hyperoxic WT female lungs (Figure 2B).

Measurements of RAC, however, showed that lack of SP-A impacts alveolar growth independent of supplemental oxygen exposure (Figure 2C) and gender (Figure 2D). The combined RAC indices of male and female mice of 3.9 ± 1.2 (Mean ± SD) and 4.0 ± 1.5 (Mean ± SD) in normoxic and hyperoxic SP-A-deficient lungs were significantly lower than 8.7 ± 2.0 (adjusted *p* < 0.0001) and 7.9 ± 2.0 (adjusted *p* < 0.0001) in WT normoxic and hyperoxic pups, respectively (Figure 2C). Alveolar growth under conditions of hyperoxia appeared to occur faster in female lungs, although differences were not statistically different (Figure 2D). This, however, reflects a significant difference in the RAC index between WT and SP-A-deficient female pups (Figure 2D).

Taken together, these results indicate that the underdeveloped SP-A-deficient lungs are vulnerable to hyperoxia-induced injury with more severe impact in the female premature lung.

### 3.2. Lack of SP-A Enhances the DNA Damage Response to Hyperoxia in Lung Macrophages

**Figure 3:**
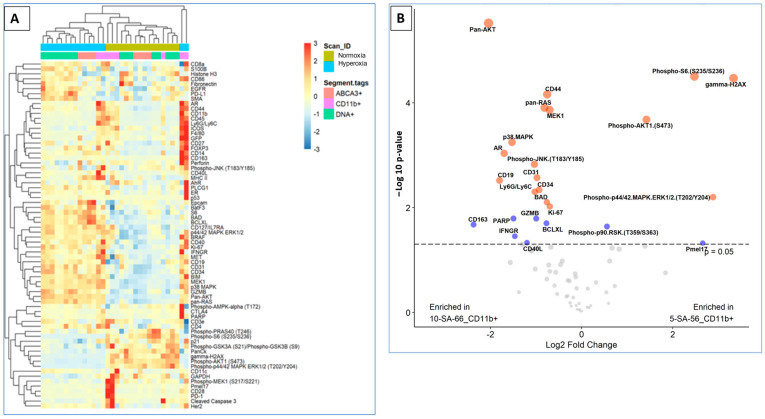
Spatial proteomic profiling of neonatal mouse lungs shows that lack of SP-A alters the response to hyperoxia and contributes to lung injury. SP-A deficient neonatal pups were exposed to either normoxia or hyperoxia with oxygen exposure at 60% from birth till postnatal day 7. (**A**) The heatmap analysis demonstrates the differential protein expression profile of hyperoxia versus normoxia among the 72 targeted proteins that were compared in different lung populations such as macrophage (CD11b+), Type II cells (ABCA3+) and DNA (**B**) The volcano plot depicts the proteins that have been upregulated and downregulated in the SP-A-deficient mouse alveolar macrophage upon exposure to hyperoxia in comparison to normoxia. The blue color dot depicts the proteins with a –log 10 *p*-value less than 2, and the red color dot shows a −log 10 *p*-value of more than 2.

**Figure 4:**
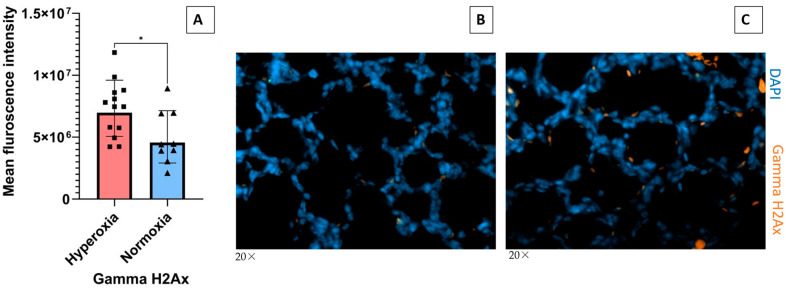
SP-A deficiency in hyperoxia-exposed mouse pups simulates alveolar injury in a mouse model of BPD. Neonatal pups were exposed to normoxia or hyperoxia (O_2_ at 60%) from birth till PND7. (**A**) Quantification of mean fluorescence intensity of γH2Ax in SP-A-deficient mouse pups exposed to normoxia and hyperoxia (one or more separate areas from each mouse formalin fixed paraffin embedded lung section was imaged and quantified, total mice n > 2 mice per group). (**B**,**C**) Immunofluorescence images of γH2Ax staining at 20× magnification (γH2Ax - orange color, DAPI nuclei - blue color) in normoxia (**B**) and hyperoxia (**C**). * *p* < 0.05.

SP-A is the principal oxidant-sensitive component and modulator of reactive oxygen species by AMs in pulmonary surfactant [11,20,21]. Here, we utilized spatial proteomics to assess whether lack of SP-A impacts the hyperoxia response in neonatal lung immune microenvironment (Figure 3). To address this question, we applied spatial proteomic profiling using GeoMx^®^ Nanostring to assess protein expression in ABCA3+ type II cells, DNA, CD11b+ macrophages in hyperoxic and normoxic lungs of SP-A-deficient mice (Figure 3A,B and Supplemental Figure S1). The cluster analysis shows the differential protein expression profile among the 72 targeted proteins after exposure to hyperoxia vs. normoxia in CD11b+ macrophages and ABAC3+ alveolar type II epithelial cells (Figure 3A). The volcano plot in Figure 3B shows a 2- and 4-fold increase in phosphorylated S6 and the formation of gamma-H2Ax in CD11b+ macrophages in SP-A-deficient lungs exposed to hyperoxia (*p* < 0.05). These results indicate that lung macrophages in SP-A-deficient mice experience increased levels of hyperoxic stress compared to normoxia. Oxidative stress via hyperoxia in SP-A KO mouse pups resulted in increased expression of γH2AX as 7.3 × 10^6^ mean intensity when compared to normoxia SP-A KO mouse pups as 4.9 × 10^6^ mean intensity (*p* = 0.026) (Figure 4). These results indicate that lung macrophages in SP-A-deficient mice experience increased levels of hyperoxic stress compared to normoxia.

## 4. Discussion

To our knowledge, we present the first known evaluation of the effects of hyperoxia on neonatal mouse pups in the absence of SP-A. Prior research involving rodent models exposed to hyperoxia by Nogee et al. observed an increase in surfactant protein A (SP-A) in rats subjected to elevated oxygen [22]. Correspondingly, another published study confirmed that, among the surfactant proteins, only SP-A exhibited an increase in response to hyperoxia [23]. Based on these findings, we hypothesized that the absence of SP-A would influence the extent of lung injury when exposed to hyperoxia. We demonstrate that SP-A-deficient neonatal mice exhibit greater arrest in alveolar development at 60% O_2_ compared to WT mice. The O_2_ dosage was shown to cause morphological changes in WT neonatal lungs following prolonged exposure from P1 to P14 days after birth [17]. Our study shows that a lack of SP-A accelerates the effect of hyperoxia on alveolar arrest within a shorter time frame of oxygen exposure. Our findings reflect those of preterm infants at risk of BPD, who may develop lung injury even with lower oxygen exposure.

Lack of SP-A reduced RAC similarly in both male and female hyperoxic lungs compared to normoxia. The RAC index of normoxic SP-A-deficient lungs, however, was lower than that of WT lungs, suggesting a novel role of SP-A in perinatal lung development. This hypomorphism may contribute to increased susceptibility to oxidant injury in the immature preterm lung when SP-A is not yet induced. Recent studies showed that SP-A supports the integrity of the developing airspace from inflammation through the SP-A receptor SP-R210 [24].

Data analysis according to gender indicates a greater impact of gender in hyperoxic female lungs of SP-A-deficient mice. Previous studies reported sexual dimorphism at a higher 95% O_2_ [25]. Biochemical assays and secretion of inflammatory mediators on day 7 in this study showed increased inflammation in females, although alveolar simplification was not quantitated on this day. Sex-dependent differences in alveolar simplification were inferred by immunohistochemical differences in endothelial cell remodeling with lesser impact in females [25]. The present results show that WT female lungs had the highest increase in RAC index under hyperoxia, consistent with a more resilient endothelium in female lungs. Lack of SP-A, however, also resulted in the highest MLI in female lungs. Adult female SP-A-deficient mice are also noted to be affected more by oxidative stress compared to their male counterparts [26]. However, clinically, males are affected more with severe BPD, and WT male mice exposed to hyperoxia were noted to have increased alveolar simplification compared to females [25]. The present results, however, indicate that SP-A-deficient males exposed to hyperoxia exhibit increased interstitial infiltration comparable to WT counterparts, indicating that sex-specific response to hyperoxia needs further exploration beyond lung morphometry. Additional studies are therefore needed to decipher the intersection of SP-A and gender in alveolar expansion, hyperoxic saccular injury, and inflammation.

Alveolar macrophages (AMs) play central roles in postnatal lung function and development [27,28,29,30,31,32,33,34,35] and are critically involved in the pathogenesis of BPD [27,36,37,38]. Preterm infant lung macrophages primed with 65% O_2_ display a persistent inflammatory response to LPS [37]. Prior studies have reported **γ**H2Ax as a response to DNA damage resulting from oxidative stress in lung tissues associated with chronic pulmonary obstructive disease [39,40] and pulmonary arterial hypertension [41]. Our preliminary spatial proteomic profiling indicating an increase in **γ**H2Ax in macrophages in hyperoxic SP-A-deficient lungs warrants additional studies to determine whether SP-A modulates the DNA damage response in lung macrophages that would indicate SP-A administration as a treatment to promote lung development in premature neonates.

Our study is limited as we did not investigate the effects of the absence of SP-A on the timing of parturition. SP-A secreted by the fetal lung induces parturition [9]. Our prior published work has also noted that SP-A affects parturition via receptor SP-R210_L_ [24]. Future studies are necessary to delineate the effect of SP-A in the setting of hyperoxia on the timing of parturition. Another limitation of our study is that the mouse model we used did not address the multifactorial nature of bronchopulmonary dysplasia (BPD). We specifically focused on oxidative stress injury in neonatal lungs. Other factors, such as the effects of steroids, infections, and chronic inflammation, were not investigated in our research, but they may also contribute to lung injury in the absence of SP-A.

In conclusion, our preliminary research indicates that the lack of SP-A modulates hyperoxia-induced lung injury. These findings call for further investigation into the mechanisms by which SP-A attenuates the development of BPD in early infancy.

## Data Availability

The data will be made available by Shaili Amatya and can be reached out at samatya@pennstatehealth.psu.edu

## Supplementary Materials

The following supporting information can be downloaded at: https://www.mdpi.com/article/10.3390/antiox13111309/s1, Figure S1: Proteomic Digital Spatial Profiling (GeoMx Nanostring) on two representative male neonatal mouse lungs exposed to hyperoxia vs. normoxia. A total of 12 regions of interest (ROI) were identified in each lung specimen with morphology markers (3 SMA, 3 DNA, 3 CD11b, 3 ABCA3).
