# Supplementary material for: Loss of Surfactant Protein A Alters Perinatal Lung Morphology and Susceptibility to Hyperoxia-Induced Bronchopulmonary Dysplasia
Source: Antioxidants (Basel). 2024 Oct 28;13(11):1309. doi: 10.3390/antiox13111309 (PMC11591242; doi:10.3390/antiox13111309)
Supplement: Supplementary file 1 [file antioxidants-13-01309-s001.zip › antioxidants-3260096-supplementary.pdf]

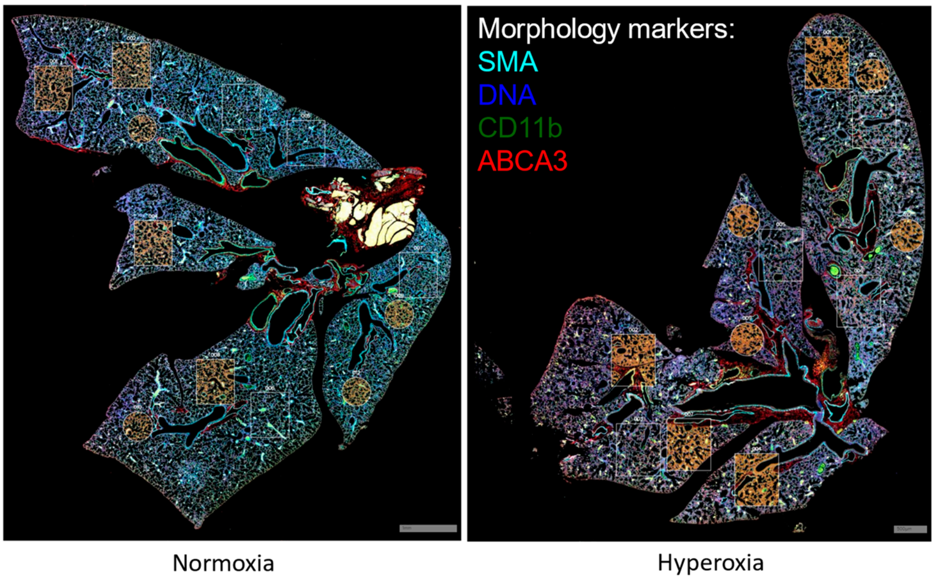

Supplementary Figure S1. Proteomic Digital Spatial Profiling (GeoMx Nanostring) on two representative male neonatal mouse lungs exposed to hyperoxia vs normoxia. 12 Regions of interest (ROI) were identified in each lung specimen with morphology markers (3 SMA, 3 DNA, 3 CD11b, 3 ABCA3).
